# Efficacy of aerobic exercise and a prudent diet for improving selected lipids and lipoproteins in adults: a meta-analysis of randomized controlled trials

**DOI:** 10.1186/1741-7015-9-74

**Published:** 2011-06-15

**Authors:** George A Kelley, Kristi S Kelley, Susan Roberts, William Haskell

**Affiliations:** 1 Department of Community Medicine, West Virginia University, PO Box 9190, Morgantown, WV 26506-9190, USA; 2 Jean Mayer Human Nutrition Research Center on Aging, Tufts University, Boston, MA, USA; 3 Prevention Research Center, Stanford University, Palo Alto, CA, USA

## Abstract

**Background:**

Studies addressing the effects of aerobic exercise and a prudent diet on lipid and lipoprotein concentrations in adults have reached conflicting conclusions. The purpose of this study was to determine the effects of aerobic exercise combined with a prudent diet on lipid and lipoprotein concentrations in adults.

**Methods:**

Studies were located by searching nine electronic databases, cross-referencing, and expert review. Two independent reviewers selected studies that met the following criteria: (1) randomized controlled trials, (2) aerobic exercise combined with diet recommendations (saturated/trans fat intake less than 10% of total calories and cholesterol less than 300 mg/day and/or fiber intake ≥25 g/day in women and ≥35 grams per day in men), (3) intervention ≥4 weeks, (4) humans ≥18 years of age, (5) published studies, including dissertations and Master's theses, (6) studies published in any language, (7) studies published between January 1, 1955 and May 1, 2009, (8) assessment of one or more of the following lipid and lipoprotein concentrations: total cholesterol (TC), high-density lipoprotein cholesterol (HDL-C), ratio of TC to HDL-C, non-HDL-C, low-density lipoprotein cholesterol (LDL-C) and triglycerides (TG). Two reviewers independently extracted all data. Random-effects models that account for heterogeneity and 95% confidence intervals were used to pool findings.

**Results:**

Of the 1,401 citations reviewed, six studies representing 16 groups (8 intervention, 8 control) and up to 559 men and women (282 intervention, 277 control) met the criteria for analysis. Statistically significant intervention minus control reductions were found for TC (-15.5 mg/dl, 95% CI, -20.3 to -10.7), TC:HDL-C (-0.4 mg/dl, 95% CI, -0.7 to -0.2), LDL-C (-9.2 mg/dl, 95% CI, -12.7 to -5.8) and TG (-10.6 mg/dl, 95% CI, -17.2 to -4.0) but not HDL-C (-0.5 mg/dl, 95% CI, -4.0 to 3.1). Changes were equivalent to reductions of 7.5%, 6.6%, 7.2% and 18.2% respectively, for TC, TC:HDL-C, LDL-C and TG. Because of missing variance statistics, non-HDL-C was excluded.

**Conclusions:**

Aerobic exercise combined with a prudent diet is highly efficacious for improving TC, TC:HDL-C, LDL-C and TG, but not HDL-C concentrations, in adults. However, additional studies are needed, including effectiveness studies using intention-to-treat analysis.

## Background

Cardiovascular disease (CVD) is a major public health problem with more than 81 million American adults (about one in three) having one or more types of CVD [1]. In terms of mortality, the estimated annual death rate from CVD in the United States was 831,272, approximately 34.3% of all deaths, in 2006 [1]. The costs associated with CVD are also high. In 2010, the annual total direct and indirect costs associated with CVD in the United States were estimated to be $503.2 billion [1]. One of the major risk factors for CVD is less than optimal lipid and lipoprotein concentrations, a common problem among American adults. In 2006, the prevalence of less than optimal concentrations of lipids and lipoproteins was estimated to be 102.2 million for total cholesterol (TC), 35.1 million for high-density lipoprotein cholesterol (HDL-C) and 71.2 million for low-density lipoproteins cholesterol (LDL-C) [1], the primary target of lipid lowering therapy in adults [2]. These prevalence rates included 46.8% of all American adults for TC, 16.2% for HDL-C and 32.6% for LDL-C [1].

Physical activity and a prudent diet, defined as a diet in which saturated/trans fat intake is less than approximately 10% of total calories and cholesterol is less than 300 mg/day and/or fiber intake is ≥25 g/day in women and ≥35 g/day in men, are low cost lifestyle changes that have been recommended for improving lipid and lipoprotein concentrations, especially LDL-C, in adults [3]. However, randomized controlled trials that have employed diets aimed at improving lipids and lipoproteins but may or may not meet the previously stated definition, have led to conflicting findings [4-19]. Meta-analysis is an approach that can increase statistical power for primary endpoints and subgroups, resolve uncertainty when studies disagree, improve estimates of treatment effect and answer questions not posed at the start of individual trials [20]. Given the conflicting findings and strengths of meta-analysis, the purpose of this study was to use the aggregate data meta-analytic approach to determine the effects of recommendations to perform aerobic exercise combined with recommendations to consume a prudent diet on lipid and lipoprotein concentrations in adults.

## Methods

### Data sources

For this proposed project, studies were located by searching nine electronic databases (PubMed, EMBASE, CINAHL, Cochrane Central Register of Controlled Trials, SportDiscus, Dissertation Abstracts International, Physiotherapy Evidence Database (PEDRO), Latin American and Caribbean Health Sciences Database (LILACS), Web of Science), cross-referencing from retrieved studies, and expert review (SR, WH). Major key words used in the electronic database searches included, but were not limited to, exercise, diet, and cholesterol. Per the recent Preferred Reporting Items for Systematic Reviews and Meta-Analyses (PRISMA) recommendations [21], the search criteria for one of the databases searched (PubMed) is shown in Additional File 1.

### Study selection

Studies were included if they met the following criteria: (1) randomized controlled trials, (2) aerobic exercise combined with diet recommendations in which saturated/trans fat intake was less than approximately 10% of total calories and cholesterol was less than 300 mg/day and/or fiber intake was ≥25 g/day in women and ≥35 g/d in men, (3) intervention ≥4 weeks, (4) humans ≥18 years of age, (5) published studies, including dissertations and Master's theses, (6) studies published in any language, (7) studies published between January 1, 1955 and May 1, 2009, (8) assessment of one or more of the following concentrations of lipids and lipoproteins: TC, HDL-C, ratio of TC to HDL-C, non-HDL-C, LDL-C, TG. The year 1955 was chosen as the starting point for potential inclusion of studies since this appeared to be the first time that an intervention on this topic had been conducted [22]. Exclusion criteria included any studies not meeting the criteria above. All studies were selected by the first two authors with discrepancies resolved by consensus as well as consultation with the last two authors if consensus could not be reached.

### Data abstraction

Prior to coding all studies, an electronic codebook was developed. The major categories that were coded included (1) study characteristics, (2) subject characteristics, (3) diet and exercise program characteristics and (4) changes in primary and secondary outcomes. All studies were coded by the first two authors, independent of each other. They then reviewed every item for accuracy and consistency. Disagreements were resolved by consensus. When consensus could not be reached, the other two authors served as arbitrators. Using Cohen's kappa statistic [23], the overall agreement rate (yes/no) prior to correcting discrepant items was 0.94.

The original study protocol included an examination of study quality using a previously developed instrument [24]. However, since the time that the original study protocol was developed, the use of quality scales has been discouraged by the Cochrane Collaboration because of the lack of empirical evidence [25,26], including validity [27], to support the use of such. Therefore, the study protocol was revised in favor of the risk of bias assessment tool recently recommended by the Cochrane Collaboration [28]. This tool assesses bias across six domains: (1) sequence generation, (2) allocation concealment, (3) blinding to group assignment, (4) incomplete outcome data, (5) selective outcome reporting, and (6) other potential bias [28]. Each domain is classified as having either a high, low, or unclear risk of bias [28]. For this study, risk of bias was limited to the primary outcomes (TC, HDL-C, ratio of TC to HDL-C, non-HDL-C, LDL-C and TG). The decision rule for blinding was that all studies were at a low risk for bias given the methods that are used to assess lipid and lipoprotein concentrations in adults. Risk of bias was also assessed with respect to whether participants had been participating in a regular program of physical activity prior to enrollment. All assessments were conducted by the first two authors, independent of each other. Both authors then met and reviewed every item for agreement. Disagreements were resolved by consensus. Using Cohen's kappa statistic [23], overall inter-rater agreement prior to correcting discrepant items was 0.72, considered to be substantial [29].

### Statistical analysis

#### Calculation of treatment effects from each study

The primary outcomes included in this meta-analysis were concentrations of TC, HDL-C, ratio of TC to HDL-C, non-HDL-C, LDL-C and TG. Effect sizes for lipid and lipoprotein variables for each group from each study were calculated by subtracting the change score in the intervention (aerobic exercise and diet) group from the change score in the control group. Variances were calculated from the pooled standard deviations of change scores in the intervention and control groups. If change score standard deviations were not available, these were calculated from 95% confidence intervals or pre and post standard deviation values according to procedures developed by Follmann et al [30]. Each effect size was then weighted by the inverse of its variance. The original metric (milligrams per deciliter) versus some type of standardized metric was used based on the belief that the former is more clinically meaningful [31].

Secondary outcomes (changes in body weight, body mass index (BMI) in kg/m^2^, waist-to-hip ratio (WHR), maximum oxygen consumption (VO_2max _ml^.^kg^-1.^min^-1^), intake of total kilocalories, carbohydrates, total fat, saturated fat, cholesterol) were calculated using the same approach as those used for lipid and lipoprotein outcomes. Insufficient data were available for pooling non-HDL-C, percent body fat, lean body mass, waist circumference, fiber and trans-fat.

#### Pooled estimates of treatment effects for primary and secondary outcomes

Random-effects models that incorporate heterogeneity into the model were used to pool all primary and secondary outcomes from each study [32]. Multiple groups from the same study were treated independently as well as after collapsing groups so that only one effect size was available from each study. If the two-tailed 95% confidence intervals generated from the models did not cross zero, results were considered to be statistically significant.

Heterogeneity was assessed using Cochran's Q statistic and an alpha value for statistical significance of 0.10 [33]. Inconsistency of effect sizes between studies was examined using an extension of the *Q *statistic, *I*^2 ^[34]. Generally, *I*^2 ^values of 25% to <50%, 50% to <75%, and ≥75% are considered to represent small, medium, and large amounts of inconsistency [34].

Potential publication bias was examined using the data imputation approach of Duval and Tweedie [35] while the influence of each study on the overall results was examined by deleting each study from the model once. Cumulative meta-analysis, ranked by year, was used to examine results over time [36]. Prediction intervals (95%) were calculated to determine treatment effects in a new trial [37,38].

Simple, random effects meta-regression (method of moments approach) was conducted to examine the association between changes in lipid and lipoprotein concentrations and age, gender, baseline lipid and lipoprotein concentrations, length of the intervention in weeks and changes in body weight [39]. For secondary outcomes, simple meta-regression was also conducted in order to examine the association between changes in dietary outcomes (total kilocalories, carbohydrates, total fat, saturated fat, cholesterol) and gender as well as whether dietary recommendations included a reduction in total intake of kilocalories. In addition, the association between changes in body weight and gender was also examined. Non-overlapping 95% confidence intervals for the slope (*β*_1_) were considered statistically significant.

Descriptive statistics were generated using PASW, version 18.0 [40], reliability statistics using Excel 2007 [41], and all meta-analytic analyses using Comprehensive Meta-Analysis, version 2.2 [39,42]. Data are reported as mean ± standard deviation (), medians (Mdn), percentages (%) and 95% confidence intervals (95% CI).

## Results

### Study characteristics

**Figure 1:**
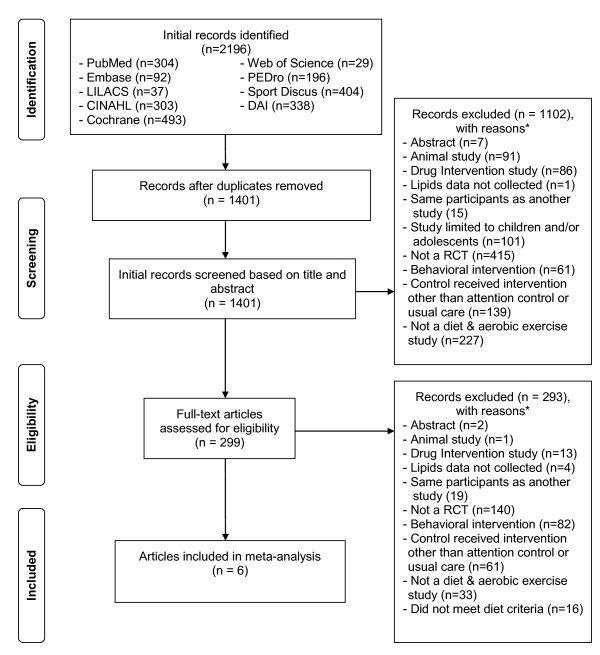
**Flow diagram for selection of articles**. *, number of reasons exceeds the number of articles because some articles were excluded for more than one reason.

A general description of the characteristics of the studies is shown in Table 1. Of the 1,401 citations reviewed, six studies representing 16 groups (8 intervention, 8 control) and up to 559 men and women (282 intervention, 277 control) met the criteria for inclusion [8,10,15,43-45]. A description of this process, including reasons for excluded studies, is shown in Figure 1. A list of excluded studies is available on request from the corresponding author. The number of intervention and control groups exceeded the number of studies because two studies reported data separately for men and women [15,45]. Dropout rates from the 4 studies and 5 groups in which data were available ranged from 0 to 10.6% in the intervention groups (, 7.1 ± 4.2, Mdn, 9) and 0 to 9.0% in the controls (, 4.2 ± 4.5, Mdn, 3) [8,43-45]. One study reported that one subject dropped out of the intervention group because they were dissatisfied with group assignment while another dropped out because of time constraints [8]. Another study reported that one subject in the control group was dropped because they failed to attend a follow-up exam [43]. The final number of participants ranged from 22 to 48 in the intervention groups (, 38 ± 9, Mdn, 41) and 22 to 46 in the control groups (, 38 ± 9, Mdn, 40). All six randomized controlled trials received some type of external funding and were published in English-language journals between 1991 and 2002 [8,10,15,43-45]. Four studies were conducted in the United States [8,10,15,45] and one each in Sweden [43] and New Zealand [44]. All of the studies appeared to use the per-protocol approach (efficacy analysis) in the treatment of their data [8,10,15,43-45]. One study reported using the Efron procedure to balance sample size, HDL-C and LDL-C levels [15] while two others stratified participants by either race (African-American versus other) [8] or gender [45]. None of the studies used a crossover design [8,10,15,43-45].

**Table 1:** Characteristics of Included Studies

Study & Year	Country	Participants^a^	Exercise	Diet	Lipid Assessment
Hellenius et al. (1993)[43]	Sweden	78 men, 35-60 yrs of age, assigned to an exercise and diet (n = 39) or control (n = 39) group	24-wks of unsupervised and supervised aerobic activity, 2-3x wk, 30-45 min/d, 60%-80% MHR	NCEP Step 1 diet (fat <30% of total kcals, saturated fat <10%, polyunsaturated fat ≤ 10%, monounsaturated fat 10%-15%, carbohydrates 50%-60%, protein 10%-20%, cholesterol <300 mg/d	TC, HDL-C, LDL-C, TG after an overnight fast and avoiding exercise for at least 24-hr
McAuley et al. (2002)[44]	New Zealand	52 men and women, 30-68 years of age assigned to an exercise and diet (n = 29) or control (n = 23) group	16-wks supervised & unsupervised aerobic exercise, 5x wk, 30 min/d	<32% of total kcals from fat, ≤ 11% of total kcals from saturated fat, 14% from monounsaturated fat, 7% from polyunsaturated fat, 50% from carbohydrates, 18% from protein, < 200 mg cholesterol and > 25 g fiber per/d	Fasting TC, HDL-C, LDL-C, TG
Miller et al. (2002)[8]	USA	43 hypertensive, overweight adults, 22 to 70 yrs of age, assigned to either an exercise and diet (n = 20) or control (n = 23) group	9-wks of supervised aerobic exercise (walking or cycle ergometry), 3x wk, 30-45 min/d, 50%-75% MHR	Hypocaloric version (2100 kcals) of DASH diet (18% kcals from protein, 55% from carbohydrate, 6% saturated fat, 13% monounsaturated fat, 8% polyunsaturated fat, 500 mg/d magnesium, 1240 mg/d calcium, 4700 mg/d potassium, 31 g/d fiber, 150 mg/d cholesterol, 100 mmol/d sodium	TC, HDL-C, LDL-C, TG after fasting
Nieman et al. (2002)[10]	USA	44 sedentary obese women, 25-75 yrs of age, assigned to either an exercise and diet (n = 22) or attention control stretching (n = 22) group	12-wks walking, 5x wk (4 supervised, 1 unsupervised), 45 min/d, 60% to 80% MHR	1200-1300 kcals, NCEP Step 1 diet (55% carbohydrate, 30% total fat, ≤ 10% saturated fat, <300 mg/d of cholesterol)	TC, HDL-C, TC/HDL-C, LDL-C, TG
Stefanick et al. (1998)[15]	USA	182 men and women, 30 to 64 yrs of age with low levels of HDL-C and high levels of LDL-C, assigned to either an exercise and diet (n = 91) or control (n = 91) group	52-wks supervised and unsupervised brisk walking and/or jogging, 60 min/d, 3x wk; goal was 10 miles brisk walking wk	NCEP Step 2 diet (< 30% total fat, <7% saturated fat, < 200 mg cholesterol/d)	TC, HDL-C, TC/HDL-C, LDL-C, TG in the morning after a 12 to 16-hr overnight fast and avoiding vigorous physical activity for at least 12 hr
Wood et al. (1991)[45]	USA	160 sedentary, overweight men and women 25 to 49 yrs of age assigned to either an exercise and diet (n = 81) or control (n = 79) group	52-wks supervised brisk walking and jogging, 25-45 min/d, 3x wk, 60% to 80% MHR	NCEP Step 1 diet (55% carbohydrate, 30% total fat, ≤ 10% saturated fat, <300 mg/d of cholesterol)	TC, HDL-C, LDL-C, TG in the morning after a 12 to 16-hr overnight fast and avoiding vigorous physical activity for at least 12 hr

NOTES: Description of groups and all other variables limited to those that met our criteria for inclusion;^a ^Number of participants limited to those in which changes in lipid and lipoprotein data were available; USA, United States of America; yrs, years; wk, week; wks, weeks; min, minutes; kcals, kilocalories; TC, total cholesterol, HDL-C, high-density lipoprotein cholesterol; LDL-C, low-density lipoprotein cholesterol; TG, triglycerides; TC:HDL-C, ratio of total cholesterol to high-density lipoprotein cholesterol; hr, hour; MHR, maximum heart rate; min/d, minutes per day; NCEP, National Cholesterol Education Program; mg/d, milligrams per day; DASH, Dietary Approaches to Stop Hypertension.

**Figure 2:**
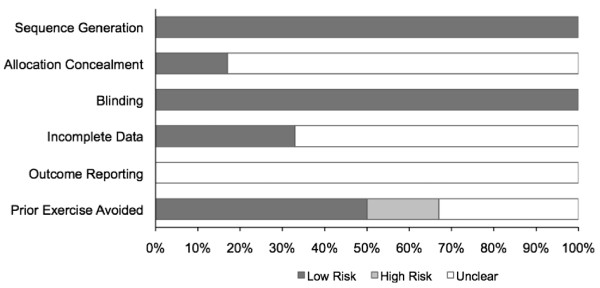
**Risk of bias assessment**.

Results for risk of bias are shown in Figure 2 and Additional File 2. Based on our assessment procedures, all of the studies were considered to be at a low risk of bias with respect to sequence generation and blinding [8,10,15,43-45]. The procedures for allocation concealment were determined to be unclear in four studies [10,15,43-45] and low in one [8]. Assessment for bias in relation to incomplete data was classified as unclear in four studies [10,15,44,45] and low in two others [8,43]. Since none of the studies provided a protocol number [8,10,15,43-45], the presence of outcome reporting bias was categorized as unclear for all of them. Potential risk of bias in relation to participation in exercise prior to study enrollment was considered to be high in one study [43], low in three [10,44,45], and unclear in the remaining two [8,15].

A description of the characteristics of the participants is shown in Table 1 and Table 2. With respect to gender, four studies included both men and women [8,15,44,45], with two studies reporting data separately for each [15,45]. Two other studies were restricted to either men [43] or women [10]. The number of men and women could not be calculated because data were not available for all studies. For the two studies that reported data on race/ethnicity, one reported that all participants were Caucasian [44] while another reported that 68% of the participants in the intervention group and 57% of participants in the control group were Black [8]. In relation to medication use of participants during the trial, two studies reported that some participants were taking some type of lipid medication prior to and during the study [8,44] while another study representing two groups reported that participants were not taking any medications known to affect blood pressure or lipid metabolism [45]. One study reported that none of the women were taking any type of hormone replacement therapy [45] while another reported that some were [15]. For cigarette smoking, two studies consisting of three groups reported that none of the participants smoked [10,45] while two others reported that some did [8,43]. Three studies reported that some of the participants consumed alcohol [8,43,44]. Participants were reported as being sedentary in three studies comprising four groups [10,44,45] while another study reported that some participants were physically active prior to enrollment [43]. One study reported no change in physical activity among control group participants during the intervention [43]. None of the studies reported whether the physical activity habits of the participants in the intervention groups changed outside of the intervention itself. For the five studies that included women [8,10,15,44,45], one each consisted of either premenopausal [45] or postmenopausal [15] women while three others included both [8,10,44]. Four studies representing five groups reported that all participants were overweight or obese [8,10,44,45] while two others representing three groups reported that some participants were overweight or obese [15,43]. With respect to hyperlipidemia, one study consisting of two groups reported that all participants were hyperlipidemic [15] while four others comprising five groups included some participants who were hyperlipidemic [8,10,43,45]. Five studies representing six groups appeared to consist of participants without type 1 or type 2 diabetes [8,10,15,43,44]. One study reported that all participants were hypertensive at study enrollment [8].

**Table 2:** Baseline characteristics of participants

	Exercise				Control			
	
Variable	#		Range	Mdn	#		Range	Mdn
Age (years)	8	47.1 ± 5.9	39-57	47.0	8	46.9 ± 6.1	39-57	45.9
Body weight (kg)	7	86.3 ± 10.6	70-98	89.9	7	88.2 ± 12.5	70-103	90.5
BMI (kg^.^m^2^)	4	31.4 ± 3.5	26-34	32.7	4	32.1 ± 5.3	25-37	33.7
WHR	4	0.91 ± 0.07	0.8-1.0	0.93	4	0.91 ± 0.07	0.8-1.0	0.93
VO_2max _(ml^.^kg^-1.^min^-1^)	5	31.3 ± 4.9	26-38	32.0	5	30.7 ± 4.9	26-38	29.0
Kilocalories (total)	6	2153 ± 305	1814-2616	2064	6	2153 ± 325	1814-2616	2090
Carbohydrates (%)	6	51 ± 5.2	44-57	52.5	6	51 ± 4.7	46-57	50.8
Total fat (%)	7	33.6 ± 3.6	28-38	33	7	33.9 ± 3.5	28-38	34.0
Saturated fat (%)	7	12.0 ± 2.1	9-14	12.4	7	12.0 ± 2.2	9-14	12.5
Cholesterol (mg)	5	282 ± 81	175-400	286	5	270 ± 85	175-400	256
TC (mg/dl)	8	218.6 ± 20.7	193-253	216.7	8	213.5 ± 16.7	193-239	209.0
HDL-C (mg/dl)	8	46.8 ± 7.6	36-58	45.5	8	45.7 ± 7.5	36-58	44.8
TC:HDL-C (mg/dl)	5	5.0 ± 1.0	3.6-6.4	4.9	5	5.2 ± 0.8	4.1-6.4	5.2
LDL-C (mg/dl)	8	145.2 ± 21.0	119-180	145.6	8	142.0 ± 15.9	119-161	141.7
TG (mg/dl)	7	86.8 ± 54.6	33-171	60.7	7	85.0 ± 55.6	33-171	60.7

Notes: #, number of groups in which data were available; With the exception of age, data limited to those in which change outcome results could be calculated; Mdn, median; BMI, body mass index; WHR, waist to hip ratio; VO_2max_, maximum oxygen consumption; mg, milligrams; TC, total cholesterol; HDL-C, high-density lipoprotein cholesterol; TC:HDL-C, ratio of TC to HDL; LDL-C, low-density lipoprotein cholesterol; TG, triglycerides; To convert TC, HDL-C and LDL-C from mg/dl to mmol, divide by 38.46; To convert TG from mg/dl to mmol, divide by 87.72; Insufficient data reported for protein, polyunsaturated fat, saturated fat, and trans-fat intake as well as non-HDL-C.

A description of the aerobic exercise component of the intervention for each study is shown in Table 1. The length of the interventions ranged from 9 to 52 weeks (, 34 ± 20, Mdn, 38). For those studies in which data were available, the between-group frequency of aerobic training ranged from 2 to 5 times per week (, 3 ± 1, Mdn, 3). Mean between-group duration of training for the three studies and four groups in which data were available [10,15,43] ranged from 45 to 60 minutes per session (, 55 ± 7, Mdn, 58). Within-group intensity of training for the five studies and six groups in which data were available [8,10,43-45] ranged from 50% to 90% of maximum heart rate (MHR). We were unable to calculate between-group statistics for intensity of training because of a lack of reported data. Mean between-group minutes of aerobic exercise for the three studies and four groups in which data could be calculated ranged from 139 to 225 minutes per week (, 181 ± 35, Mdn, 180) [10,15,43]. As can be seen in Table 1, walking was the most common form of aerobic exercise prescribed. Compliance for the two studies that reported such was 86% for one study [8] and 95% for the other [10]. Compliance appeared to be assessed by the investigator in the first study [8] and by a combination of investigator and self-reported assessment in the other [10]. Four studies representing five groups had participants perform both supervised and unsupervised exercise [10,15,43,44] while two studies consisting of three groups had participants perform supervised exercise only [8,45]. All supervised exercise appeared to be facility-based while unsupervised exercise appeared to be home-based.

A description of the diet component of the intervention for each study is shown in Table 1. Five studies involved the provision of dietary recommendations [10,15,43-45] while one study provided food [8]. Three studies representing four groups used the National Cholesterol Education Program (NCEP) Step 1 diet [10,43,45], one study comprised of two groups used the NCEP Step 2 diet [15] while another used the Dietary Approaches to Stop Hypertension (DASH) diet [8]. Intentional weight loss was a component of the diet intervention for all participants in three studies comprised of four groups [8,10,45] and for only those participants who were overweight or obese in two other studies [43,44]. For these studies, kilocalories (kcals) consumed per day was limited to 2,100 in one study [8] and 1,200 to 1,300 in another [10]. Two other studies reported that kcals consumed per day were individually-based [43,44]. For the five studies that reported information on the assessment of nutrition intake [10,15,43-45], two studies representing three groups reported using self-reported seven day food records [43,45] with one reporting that food records were both self-reported and interviewer administered [45]. One study each reported the assessment of nutrition intake using either a three day food record and 24 hour recall [10], four day diet record [44], or four weekdays and one weekend day from 24 hour dietary recall via telephone interviews [10].

Results for the assessment of lipid and lipoprotein concentrations are shown in Table 1. All but one study [10] reported that participants fasted prior to lipid assessment with fasting occurring for at least 12 hours. Three studies also reported that exercise was avoided for at least 12 hours prior to lipid assessment [15,43,45]. All reported that lipid and lipoprotein assessments appeared to take place in the morning with one study reporting duplicate measures [15]. Four studies reported using the Friedwald formula for estimating LDL-C [8,10,15,45]. Insufficient information was provided to determine if lipid and lipoprotein concentrations were assessed during the same season [8,10,15,43-45].

For those studies in which information was available, assessment of body weight occurred using a standard balance beam scale in three studies comprising five groups [8,15,45] while another reported using a calibrated electronic scale [44]. Two studies representing three groups reported that participants wore light clothing without shoes during body weight assessment [8,45]. One study consisting of two groups reported that body weight was assessed during two visits [15]. Body mass index was calculated from measurements of height and weight. With respect to the assessment of waist to hip ratio, two studies representing three groups reported assessment with a tape measure at the narrowest part of the waist and the widest part of the hips [15,43]. Three studies representing five groups reported the assessment of VO_2max _in ml^.^kg^-1.^min^-1 ^using a treadmill [15,44,45]. Two studies consisting of four groups appeared to have participants exercise to volitional fatigue [15,45] while the third study estimated VO_2max_ from a submaximal treadmill test (Bruce protocol) [44].

### Outcomes

#### Primary outcomes (changes in lipids and lipoproteins)

**Figure 3:**
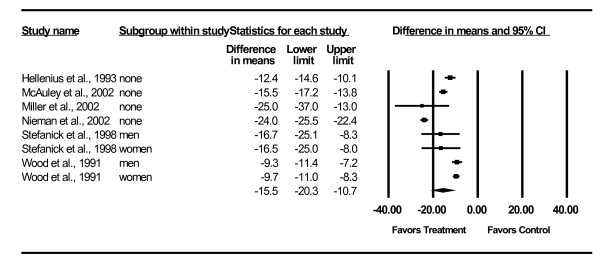
**Forest plot for changes in TC**.

**Figure 4:**
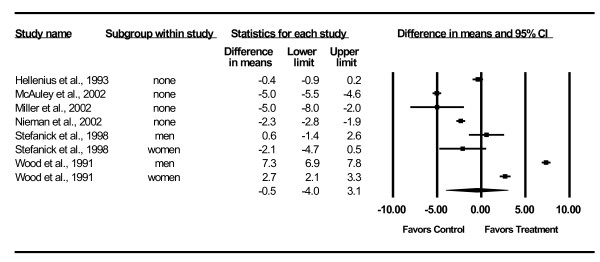
**Forest plot for changes in HDL-C**.

**Figure 5:**
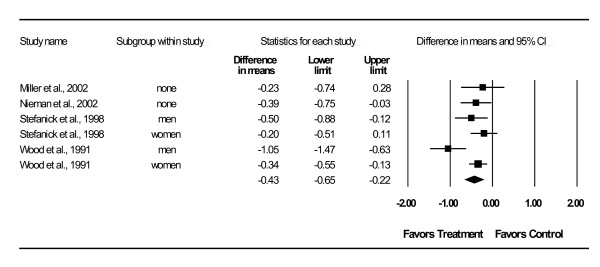
**Forest plot for changes in TC:HDL-C**.

**Figure 6:**
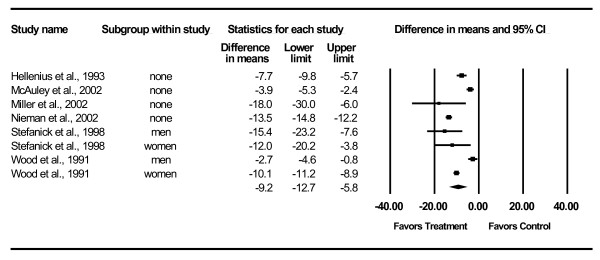
**Forest plot for changes in LDL-C**.

**Figure 7:**
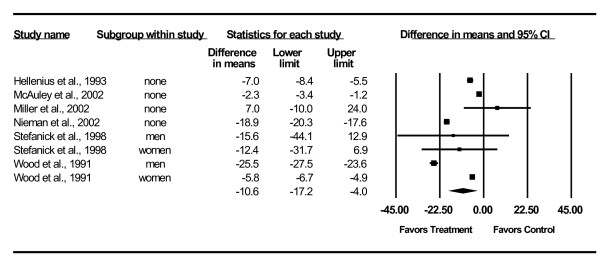
**Forest plot for changes in TG**.

Changes in lipid and lipoprotein concentrations are shown in Table 3 and Figures 3,4,5,6,7. As can be seen, statistically significant intervention minus control reductions were found for TC, ratio of TC to HDL-C, LDL-C, and TG but not for HDL-C. Changes in non-HDL-C could not be calculated because none of the studies reported variance statistics for such. None of the studies reported changes in non-HDL-C. Changes were equivalent to intervention minus control reductions of 7.5%, 6.6%, 7.2% and 18.2%, respectively, for TC, ratio of TC to HDL-C, LDL-C and TG. Significant heterogeneity and inconsistency as well as overlapping prediction intervals were found for all lipid and lipoprotein concentrations. With each study deleted from the model once, results remained statistically significant for TC, ratio of TC to HDL-C, LDL-C, and TG while HDL-C remained non-significant [see Additional Files 3,4,5,6,7]. When adjusted for publication bias, results remained statistically significant for TC (-14.7 mg/dl, 95% CI, -19.3, -10.1) and LDL-C (-8.0 mg/dl, 95% CI, -11.3, -4.8). No adjustment for publication bias was necessary for HDL-C, ratio of TC to HDL-C, LDL-C or TG. Cumulative meta-analysis ranked by year, showed that results have remained non-significant for HDL-C and significant for TC, ratio of TC to HDL-C, LDL-C and TG since at least 1998 [see Additional Files 8,9,10,11,12]. The direction of results did not change when multiple groups from the same studies were collapsed so there was only one effect size from each study (TC,-16.5 mg/dl, 95% CI, -22.3, -10.8; HDL-C, -1.2 mg/dl, 95% CI, -5.3, 2.8; ratio of TC to HDL-C, -0.4, 95% CI, -0.5, -0.3; LDL-C, -9.6 mg/dl, 95% CI, -13.2, -6.1; TG, -8.4 mg/dl, 95% CI, -14.1, -2.8).

**Table 3:** Changes in primary and secondary outcomes

Variable	Studies (#)	Participants (#)	ES (#)	(95% CI)	Q(p)	***I^2^(%)***	95% PI
Primary Outcomes							
- TC (mg/dl)	6	559	8	-15.5 (-20.3, -10.7)^a^	221.0 (<0.001)^a^	96.8	-32.1, 1.1
- HDL-C (mg/dl)	6	559	8	-0.5 (-4.0, 3.1)	1678.9 (<0.001)^a^	99.6	-13.6, 12.6
- TC:HDL-C	4	429	6	-0.4 (-0.7, -0.2)^a^	11.5 (0.04)^a^	56.7	-1.0, 0.1
- LDL-C (mg/dl)	6	559	8	-9.2 (-12.7, -5.8)^a^	143.5 (<0.001)^a^	95.1	-21, 2.5
- TG (mg/dl)	6	559	8	-10.6 (-17.2, -4.0)^a^	691.9 (<0.001)^a^	99.0	-32.5, 11.3
Secondary Outcomes							
- Body weight (kg)	5	481	7	-5.7 (-7.4, -4.1)^a^	26.7 (<0.001)^a^	77.5	-11.0, -0.5
- BMI (kg/m^2^)	4	217	4	-1.5 (-2.1, -0.8)^a^	11.7 (0.008)^a^	74.3	-4.2, 1.3
- WHR	3	312	4	-0.01(-0.015, 0.003)	4.0 (0.3)	25.8	-0.03, 0.02
- VO_2max_(ml^.^kg^-1.^min^-1^)	4	384	5	5.1 (2.7, 7.5)^a^	33.2 (<0.001)^a^	88.0	-3.9, 14.1
- Kilocalories (total)	4	450	6	-283 (-453, -114)^a^	17.5 (0.004)^a^	71.5	-805, 238
- Carbohydrates (%)	4	323	5	6.0 (4.0, 8.0)^a^	18.6 (0.001)^a^	78.4	-1.1, 13.2
- Total fat (%)	5	493	7	-7.6 (-9.8, -5.3)^a^	54.1 (<0.001)^a^	88.9	-15.3, 0.2
- Saturated fat (%)	4	348	5	-4.2 (-5.4, -3.1)^a^	24.3 (<0.001)^a^	83.6	-8.5, 0.1
- Cholesterol (mg)	3	386	5	-115 (-148, -83)^a^	10.3 (<0.04)^a^	61.2	-222, -9.0

Notes: ES, effect sizes; #, number in which data were available for; (95% CI), mean and 95% confidence interval; Q(p), Cochran's Q statistic for heterogeneity and alpha value; *I^2 ^(%)*, percent inconsistency statistic; 95% PI, 95% prediction intervals for a new study; ^a^statistically significant; TC, total cholesterol; HDL-C, high-density lipoprotein cholesterol; TC:HDL-C, TC:HDL-C ratio; LDL-C, low-density lipoprotein cholesterol; TG, triglycerides; BMI, body mass index; WHR, waist to hip ratio; VO_2max_, maximum oxygen consumption; %, percentage of total kilocalorie intake; To convert TC, HDL-C and LDL-C from mg/dl to mmol, divide by 38.46; To convert TG from mg/dl to mmol, divide by 87.72.

Meta-regression analyses resulted in several significant associations. For TC, shorter interventions were associated with greater reductions in TC (*β*_1_, 0.21, 95% CI, 0.03, 0.38) while greater increases in HDL-C were associated with younger age (*β*_1_, -0.51, 95% CI, -0.90, -0.13), longer interventions (*β*_1_, 0.16, 95% CI, 0.05, 0.26) and greater decreases in body weight (*β*_1_, -1.44, 95% CI, -2.5, -0.37). Greater decreases in the ratio of TC:HDL-C were associated with being male (*β*_1_, -0.44, 95% CI, -0.82, -0.06) and greater decreases in body weight (*β*_1_, 0.10, 95% CI, 0.03, 0.18). Greater decreases in TG were also associated with larger decreases in body weight (*β*_1_, 3.20, 95% CI, 1.05, 5.35). No other significant associations were observed for any of our lipid and lipoprotein concentrations.

#### Secondary outcomes

Results for secondary outcomes are shown in Table 3. A statistically significant intervention minus control reduction was found for body weight and BMI but not for WHR. Changes were equivalent to intervention minus control reductions of 6.7%, 5.0% and 0.5%, respectively for body weight, BMI and WHR. Statistically significant heterogeneity as well as inconsistency was observed for body weight and BMI but not for WHR. Overlapping prediction intervals were observed for BMI and WHR but not for body weight. For VO_2max _in ml^.^kg^-1.^min^-1^, statistically significant intervention minus control increases equivalent to 16.3% were found. Statistically significant heterogeneity and/or inconsistency as well as overlapping prediction intervals were also identified. There was no significant association between changes in body weight and gender (*β*_1_, -1.6, 95% CI, -6.9, 3.6).

For daily nutrition intake, statistically significant intervention minus control reductions were observed for total kilocalories, percentage of kilocalories from total fat, percentage of kilocalories from saturated fat, and daily cholesterol consumption. In addition, there was a statistically significant increase in the percentage of carbohydrates consumed. Across all studies, changes were equivalent to intervention minus control improvements of 14.9%, 12.0%, 23.1%, 34.7% and 42.1% respectively, for total kilocalories, carbohydrates, total fat, saturated fat and cholesterol. With the exception of prediction intervals for cholesterol intake, statistically significant heterogeneity and inconsistency as well as overlapping prediction intervals were observed for all nutrition outcomes.

Greater reductions in dietary outcomes were associated with studies that recommended lower caloric intake for total kilocalories (*β*_1_, -310, 95% CI, -475, -146) and percentage of carbohydrates (*β*_1_, -3.0, 95% CI, -5.7, -0.3) but not for percentage of total fat (*β*_1_, -0.4, 95% CI, -2.8, 2.0), percentage of saturated fat (*β*_1_, 0.05, 95% CI, -0.9, 1.0) or cholesterol consumed (*β*_1_, -.45.9, 95% CI, -104.9, 12.5). In addition, there were no statistically significant associations between changes in dietary outcomes and gender (total kilocalories, *β*_1_, 179.1, 95% CI, -149, 507; percentage of total fat, *β*_1_, 0.9, 95% CI, -3.8, 5.6; percentage of saturated fat, *β*_1_, -0.7, 95% CI, -1.7, 0.3; cholesterol *β*_1_, -41.0, 95% CI, -108.8, 26.9; percentage of carbohydrates *β*_1_, -.1.1 95% CI, -6.9, 4.7).

## Discussion

The purpose of this study was to determine the combined effects of aerobic exercise and a prudent diet on lipid and lipoprotein concentrations in adults. The overall results suggest that the combined effects of both are efficacious for reducing concentrations of TC, the ratio of TC to HDL-C, LDL-C and TG, but not increasing HDL-C, in adults. These findings appear to be important from a practical perspective, especially in relation to the prevention of coronary heart disease (CHD), a disease with an annual estimated incidence rate in the United States of more than 1.4 million people [1]. For example, it has been reported that every 1% reduction in population levels of TC results in an approximate 2% reduction in the rate of CHD [46]. Based on the findings of the current study, this would result in a 15% reduction in the rate of CHD as a result of aerobic exercise and a prudent diet. The decreases observed in the ratio of TC to HDL-C also appear to be important with changes based on previous research equivalent to reductions of approximately 21% in CHD risk [47]. In addition, Wilson et al [48], estimated that every 1% population decrease in LDL-C would decrease the 12-year incidence of CHD by 1%. Congruent with the changes observed for LDL-C in the current meta-analysis, this would be equivalent to a reduction of approximately 7% in the 12-year incidence of CHD. Improvements in LDL-C may be particularly important given that LDL-C is currently the primary target of lipid-lowering therapy in adults [2]. Furthermore, based on previous work, the decreases in serum TG observed in the current investigation would be equivalent to decreases in the relative risk of CHD of 2% in men and 4% in women [49]. Finally, the observed results may be an underestimate of the true effects of aerobic exercise combined with a prudent diet on lipid and lipoprotein concentrations in adults given that participants tend to overestimate their adherence to exercise and diet recommendations [50,51].

The changes observed in TC, LDL-C and TG in the current investigation appear to be greater than those achieved with aerobic exercise alone. For example, previous meta-analytic work addressing the effects of aerobic exercise on lipid and lipoprotein concentrations in women [52] and men [53] yielded significant reductions in TC, LDL-C and TG that were less than half those observed in the current investigation. The ratio of TC to HDL-C was not assessed in either study [52,53]. In contrast, prior meta-analytic work that examined the effects of Step 1 and 2 diets from the American Heart Association reported improvements in lipid and lipoprotein concentrations that were similar to or larger than the current investigation with respect to TC, ratio of TC to HDL-C and LDL-C [54]. However, changes in TG were less in this prior work [54]. The results of the prior meta-analysis should be viewed with caution as it appears that traditional statistical approaches versus those specific to the conduct of meta-analysis were used [54]. Consequently, the reported changes in lipid and lipoprotein concentrations may be exaggerated.

While statistically significant and practically important improvements were observed for TC, the ratio of TC to HDL-C, LDL-C and TG, no such differences were observed for HDL-C. The lack of effect on HDL-C may not be surprising given that aerobic exercise alone has been shown to increase concentrations of HDL-C in both women [52] and men [53] while low total and saturated fat diets have been shown to significantly decrease HDL-C [54]. Thus, it appears that the positive effects of aerobic exercise on HDL-C may not override the lowering effect of diets generally low in total and saturated fat but may help to mitigate these changes given that the current meta-analysis found a non-significant decrease in HDL-C of 1.3% while low-fat only diets resulted in a statistically significant decrease of 7%. Given that participants with low concentrations of HDL-C may respond less to exercise than those with high concentrations, it is important to note that results remained consistent when each study was excluded from the model once, including the one study that enrolled participants with initially low concentrations of HDL-C [15]. With the former in mind, other forms of therapy, for example, fibrates or niacin [55], may be necessary for raising HDL-C in adults who exercise aerobically and consume a low-fat diet. However, as previously noted, the results of the low-fat diet meta-analysis may have been exaggerated [54]. Based on this observation, it appears that a need exists for an updated meta-analysis of randomized controlled trials to determine the effects of low-fat only diets on lipid and lipoprotein concentrations, especially HDL-C, in adults.

The changes observed in the current study for TC, LDL-C and HDL-C are generally less than those reported for statin therapy and equal to or greater than changes reported for TG [56]. For example, previous meta-analytic research on the effects of statins on lipid and lipoprotein concentrations reported improvements ranging from 17% to 31%, 7% to 12%, 26% to 46% and 10% to 18% respectively, for TC, HDL-C, LDL-C and TG [56]. Generally speaking, our findings support current recommendations regarding the use of aerobic exercise and a prudent diet as a first line strategy for maintaining optimal concentrations of lipids and lipoproteins in adults [2]. If optimal levels cannot be attained, aerobic exercise and diet should still be recommended with the possible addition of fibrates or niacin [55] for increasing HDL-C and/or a statin for improving all other lipid and lipoprotein concentrations, especially LDL-C [56].

Statistically significant reductions were found for body weight and BMI while changes in the direction of benefit were observed for WHR as a result of the aerobic exercise and diet intervention. In addition, a statistically significant increase in VO_2max _in ml.kg^-1.^min^-1 ^was observed. These findings are not surprising given that weight loss and increases in cardiorespiratory fitness are common changes that occur as a result of aerobic exercise and a prudent diet. Given that significant reductions were found for total kilocalories, total fat, saturated fat and cholesterol, it appears, overall, that participants were successful in adhering to the assigned diet.

While the results of this study are encouraging, they must be viewed with respect to several issues. First, while simple meta-regression analyses yielded several significant associations with selected concentrations of lipids and lipoproteins, these should be viewed with caution given the small sample size. In addition, since studies are not randomly assigned to predictors, such analyses are considered to be observational in nature [57]. Consequently, such analyses do not support causal inferences [57]. Rather, the validity of these findings would need to be tested in large, well-designed randomized controlled trials. This may be especially relevant for determining whether changes in selected lipid and lipoprotein concentrations are the result of the combined effects of aerobic exercise and a prudent diet or the weight loss associated with a combination of aerobic exercise and a prudent diet.

Second, with the exception of the WHR, statistically significant heterogeneity and a large amount of inconsistency was observed for all of our primary and secondary outcomes. While random effects models account for heterogeneity, resulting in narrower confidence intervals when heterogeneity is present, others have suggested that it is not appropriate to reach conclusions based on aggregate findings when there is significant heterogeneity and/or inconsistency [28]. However, heterogeneity (*Q) *and inconsistency (*I^2^) *statistics do not guarantee that the dispersion in results are large enough to be of practical or theoretical importance [58]. In addition, no significant differences were found for any of our outcomes when each study was deleted from the model once as well as when selected results were adjusted for publication bias. Furthermore, based on our cumulative meta-analyses, the direction of results for all of our outcomes has remained stable for more than a decade.

A third issue has to do with the fact that overlapping prediction intervals were observed for all outcomes except changes in body weight and cholesterol intake. From a practical perspective, prediction intervals may be more relevant since they provide an approximation of the expected treatment effect in a new trial [37,38]. However, they should not be used to determine whether confidence intervals from a random effects model are correct or incorrect since prediction intervals are based on a random mean effect while confidence intervals are not [37,38].

A fourth issue has to do with the lack of studies available after the investigative team's strict inclusion criteria were applied. This was especially surprising given the fact that aerobic exercise and a diet low in total and saturated fat are common first line strategies for treating dyslipidemia [2,3]. In addition, it was surprising to see that the most recent study that met our strict inclusion criteria had been published in 2002 [8,10,44]. While this may have been the result of possible search error, there may also be a general belief that the beneficial effects of this intervention are well established and that no further research in this area is necessary. However, this may be shortsighted. For example, all of the included studies appeared to conduct 'as treated' analyses in determining the combined effects of aerobic exercise and a prudent diet on lipid and lipoprotein concentrations in adults. While such an approach can determine the efficacy of findings, it cannot determine the effectiveness of findings [59]. Therefore, it would appear plausible to suggest that future studies are needed that include both efficacy (as-treated) and effectiveness (intention-to-treat) analyses and that such studies focus on the Therapeutic Lifestyle Changes currently recommended by the National Cholesterol Education Program and American Heart Association [2,3]. Such knowledge would address the issue of whether the currently recommended treatment works (efficacy) as well as whether it works in the real world (effectiveness*) *[59]. Knowledge of both is especially important with respect to the allocation of resources for treating dyslipidemia.

A fifth issue has to do with the general lack of reporting for certain information in the included studies. For example, it was difficult to assess the risk of bias with respect to allocation concealment, incomplete data and outcome reporting. It is suggested that future studies report this information, including a study protocol identification number. Future studies should also ensure that all participants were not exercising on a regular basis prior to enrollment and include a definition of such. In addition, future research on this topic should include data on the race/ethnicity of subjects, number of men and women who started and completed the study, medication status of participants, including hormone replacement therapy, cigarette smoking, alcohol intake, percentage of body fat as an outcome variable, average intensity of training for aerobic exercise, compliance to the aerobic exercise protocol, whether the exercise habits of participants changed outside of the intervention, protein, fiber, and trans-fat intake as well as the season(s) in which lipids and lipoproteins were assessed and the number of hours that exercise was avoided prior to assessment. Finally, since non-HDL-C has been shown to be a better predictor of cardiovascular morbidity and mortality than LDL-C [60,61], currently, the primary target of lipid lowering therapy in adults [2], the inclusion of data for non-HDL-C, including dispersion statistics, is also recommended in future studies.

A sixth issue has to do with the use of traditional meta-analytic models that were employed in the current meta-analysis. While more recent meta-analytic methods have been proposed [62-68], it was the investigative team's position that (1) it would be imprudent to overreact to newly proposed models and (2) that many of the newer alternatives proposed as a means of dealing with specific problems in meta-analysis may have problems of their own, possibly with substantially worse impact than the problems they were intended to solve (Dr. Michael Borenstein and Dr. Larry Hedges, personal communication, 22 February and 25 February 2011).

Finally, the focus of the current meta-analysis was on the combined effects of aerobic exercise and a prudent diet on lipid and lipoprotein concentrations in adults. However, there may also be an interest in future meta-analytic work aimed at determining the independent effects of aerobic exercise, dietary fat reduction and weight loss on lipid and lipoprotein concentrations in adults. Ideally, this would best be accomplished by limiting the inclusion of studies to randomized controlled trials in which all of these intervention arms are included within the same study.

In conclusion, aerobic exercise and a prudent diet are efficacious for improving TC, TC:HDL-C, LDL-C and TG, but not HDL-C concentrations, in adults. However, a need exists for additional studies on this topic, including effectiveness studies using intention-to-treat analysis.

## Competing interests

The authors declare that they have no competing interests.

## Authors' contributions

GAK was responsible for the conception and design, acquisition of data, analysis and interpretation of data, drafting the initial manuscript and revising it critically for important intellectual content. KSK was responsible for the conception and design, acquisition of data, and reviewing all drafts of the manuscript. SR was responsible for the conception and design, interpretation of data and reviewing all drafts of the manuscript. WH was responsible for the conception and design, interpretation of data and reviewing all drafts of the manuscript. All authors read and approved the final manuscript.

## Author information

GAK has more than 15 years of successful experience in the design and conduct of all aspects of meta-analysis, particularly as it pertains to the effects of chronic exercise on cardiovascular disease risk factors. KSK has more than 12 years of successful experience in conducting meta-analysis, particularly as it pertains to the effects of chronic exercise on cardiovascular disease risk factors. WH is a leading authority on the effects of exercise on lipids and lipoproteins in adults. SR is a leading authority on the effects of diet on lipids and lipoproteins in adults.

## Pre-publication history

The pre-publication history for this paper can be accessed here:

http://www.biomedcentral.com/1741-7015/9/74/prepub

## Supplementary Material

Additional file 1 **PubMed User Query**. This additional file contains the user query used for our electronic database search in PubMed. Click here for file

Additional file 2 **Study-level risk of bias assessment**. This additional table contains the results for risk of bias assessment at the study level. Click here for file

Additional file 3 **Sensitivity analysis for changes in TC**. This additional figure contains the results for changes in TC with each study deleted from the model once. Click here for file

Additional file 4 **Sensitivity analysis for changes in HDL-C**. This additional figure contains the results for changes in HDL-C with each study deleted from the model once. Click here for file

Additional file 5 **Sensitivity analysis for changes in TC:HDL-C**. This additional figure contains the results for changes in the ratio of TC:HDL-C with each study deleted from the model once. Click here for file

Additional file 6 **Sensitivity analysis for changes in LDL-C**. This additional figure contains the results for changes in LDL-C with each study deleted from the model once. Click here for file

Additional file 7 **Sensitivity analysis for changes in TG**. This additional figure contains the results for changes in TG with each study deleted from the model once. Click here for file

Additional file 8 **Cumulative meta-analysis for changes in TC**. This additional figure contains the cumulative results, ranked by year, for changes in TC. Click here for file

Additional file 9 **Cumulative meta-analysis for changes in HDL-C**. This additional figure contains the cumulative results, ranked by year, for changes in HDL-C Click here for file

Additional file 10 **Cumulative meta-analysis for changes in the ratio of TC:HDL-C**. This additional figure contains the cumulative results, ranked by year, for changes in the ratio of TC:HDL-C. Click here for file

Additional file 11 **Cumulative meta-analysis for changes in LDL-C**. This additional figure contains the cumulative results, ranked by year, for changes in LDL-C. Click here for file

Additional file 12 **Cumulative meta-analysis for changes in TG**. This additional figure contains the cumulative results, ranked by year, for changes in TG. Click here for file
