# Supplementary material for: Efficacy of aerobic exercise and a prudent diet for improving selected lipids and lipoproteins in adults: a meta-analysis of randomized controlled trials
Source: BMC Med. 2011 Jun 15;9:74. doi: 10.1186/1741-7015-9-74 (PMC3141539; doi:10.1186/1741-7015-9-74)
Supplement: Additional file 1 — PubMed User Query. This additional file contains the user query used for our electronic database search in PubMed. [file 1741-7015-9-74-S1.DOC]

Supplement 1. User Query for PubMed Search

("1955/01/01"[PDAT] : "2009/05/01"[PDAT]) AND (("exercise"[MeSH Terms] OR "exercise"[All Fields]) AND ("diet"[MeSH Terms] OR "diet"[All Fields]) AND ("lipids"[MeSH Terms] OR "lipids"[All Fields]) OR ("exercise"[MeSH Terms] OR "exercise"[All Fields]) AND ("diet"[MeSH Terms] OR "diet"[All Fields]) AND ("cholesterol"[MeSH Terms] OR "cholesterol"[All Fields])) AND ("humans"[MeSH Terms] AND Randomized Controlled Trial[ptyp] AND "adult"[MeSH Terms])
