# Supplementary material for: Efficacy of aerobic exercise and a prudent diet for improving selected lipids and lipoproteins in adults: a meta-analysis of randomized controlled trials
Source: BMC Med. 2011 Jun 15;9:74. doi: 10.1186/1741-7015-9-74 (PMC3141539; doi:10.1186/1741-7015-9-74)
Supplement: Additional file 2 — Study-level risk of bias assessment. This additional table contains the results for risk of bias assessment at the study level. [file 1741-7015-9-74-S2.DOC]

Supplement 2. Study level risk of bias assessment table.

| Reference | Sequence  Generation | Allocation  Concealment | Blinding | Incomplete  Data | Outcome  Reporting | Exercise  Avoided |
| --- | --- | --- | --- | --- | --- | --- |
| Hellenius et al. (1993) | Low | Unclear | Low | Low | Unclear | High |
| McAuley et al.(2002) | Low | Unclear | Low | Unclear | Unclear | Low |
| Miller et al. (2002) | Low | Low | Low | Low | Unclear | Unclear |
| Nieman et al. (2002) | Low | Unclear | Low | Unclear | Unclear | Low |
| Stefanick et al. (1998) | Low | Unclear | Low | Unclear | Unclear | Unclear |
| Wood et al. (1991) | Low | Unclear | Low | Unclear | Unclear | Low |

Notes: Low, low risk of bias that is unlikely to seriously alter the results; high, high risk of bias that could seriously weaken confidence in the results; unclear, unclear risk of bias that might affect the results (this category includes studies in which insufficient data were provided to make a judgment of high risk or low risk).
