# Supplementary material for: Efficacy of aerobic exercise and a prudent diet for improving selected lipids and lipoproteins in adults: a meta-analysis of randomized controlled trials
Source: BMC Med. 2011 Jun 15;9:74. doi: 10.1186/1741-7015-9-74 (PMC3141539; doi:10.1186/1741-7015-9-74)

Study name	Subgroup within study	Cumulative statistics		
		**Point**	**Lower limit**	**Upper limit**
Wood et al., 1991	men	-1.05	-1.47	-0.63
Wood et al., 1991	women	-0.67	-1.36	0.02
Stefanick et al., 1998	men	-0.60	-1.00	-0.20
Stefanick et al., 1998	women	-0.49	-0.80	-0.18
Miller et al., 2002	none	-0.45	-0.71	-0.18
Nieman et al., 2002	none	-0.43	-0.65	-0.22
		-0.43	-0.65	-0.22

Cumulative difference in means (95% CI)

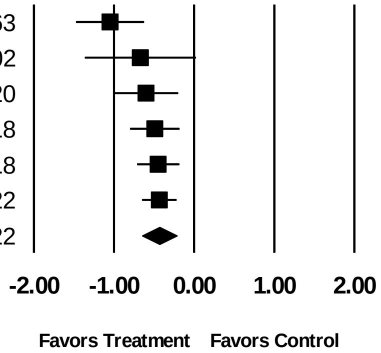

Supplement: Supplementary file 3 [file 1741-7015-9-74-S10.DOC]
