# Supplementary material for: Efficacy of aerobic exercise and a prudent diet for improving selected lipids and lipoproteins in adults: a meta-analysis of randomized controlled trials
Source: BMC Med. 2011 Jun 15;9:74. doi: 10.1186/1741-7015-9-74 (PMC3141539; doi:10.1186/1741-7015-9-74)

Study name	Subgroup within study	Cumulative statistics: Point	Lower limit	Upper limit
Wood et al., 1991	men	-2.7	-4.6	-0.8
Wood et al., 1991	women	-6.4	-13.6	0.8
Hellenius et al., 1993	none	-6.9	-11.3	-2.4
Stefanick et al., 1998	men	-8.1	-12.3	-4.0
Stefanick et al., 1998	women	-8.6	-12.4	-4.7
McAuley et al., 2002	none	-7.6	-10.9	-4.2
Miller et al., 2002	none	-8.2	-11.4	-4.9
Nieman et al., 2002	none	-9.2	-12.7	-5.8
		-9.2	-12.7	-5.8

Cumulative difference in means (95% CI)

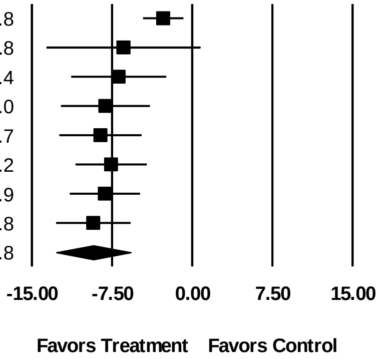

Supplement: Supplementary file 4 [file 1741-7015-9-74-S11.DOC]
