# Supplementary material for: Efficacy of aerobic exercise and a prudent diet for improving selected lipids and lipoproteins in adults: a meta-analysis of randomized controlled trials
Source: BMC Med. 2011 Jun 15;9:74. doi: 10.1186/1741-7015-9-74 (PMC3141539; doi:10.1186/1741-7015-9-74)

Study name	Subgroup within study	Cumulative statistics		
		**Point**	**Lower limit**	**Upper limit**
Wood et al., 1991	men	-25.5	-27.5	-23.6
Wood et al., 1991	women	-15.6	-35.0	3.7
Hellenius et al., 1993	none	-12.7	-22.9	-2.5
Stefanick et al., 1998	men	-13.0	-22.7	-3.2
Stefanick et al., 1998	women	-12.9	-22.0	-3.8
McAuley et al., 2002	none	-10.5	-17.4	-3.7
Miller et al., 2002	none	-9.0	-15.6	-2.5
Nieman et al., 2002	none	-10.6	-17.2	-4.0
		-10.6	-17.2	-4.0

Cumulative difference in means (95% CI)

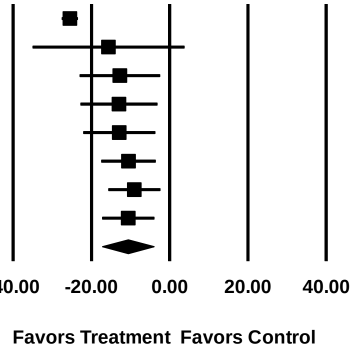

Supplement: Supplementary file 5 [file 1741-7015-9-74-S12.DOC]
