# Supplementary material for: Efficacy of aerobic exercise and a prudent diet for improving selected lipids and lipoproteins in adults: a meta-analysis of randomized controlled trials
Source: BMC Med. 2011 Jun 15;9:74. doi: 10.1186/1741-7015-9-74 (PMC3141539; doi:10.1186/1741-7015-9-74)

Study name	Subgroup within study	Point	Lower limit	Upper limit
Hellenius et al., 1993	none	-16.1	-21.6	-10.5
McAuley et al., 2002	none	-15.6	-21.5	-9.7
Miller et al., 2002	none	-14.7	-19.7	-9.7
Nieman et al., 2002	none	-13.0	-15.8	-10.2
Stefanick et al., 1998	men	-15.4	-20.4	-10.3
Stefanick et al., 1998	women	-15.4	-20.5	-10.3
Wood et al., 1991	men	-16.6	-21.9	-11.2
Wood et al., 1991	women	-16.5	-21.7	-11.3
		-15.5	-20.3	-10.7

Difference in means (95% CI) with study removed

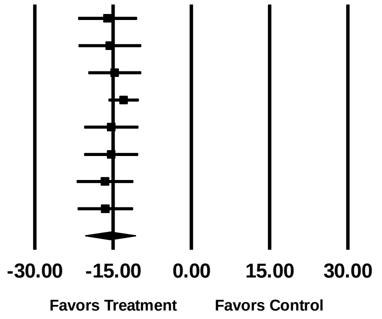

Supplement: Supplementary file 6 [file 1741-7015-9-74-S3.DOC]
