# Supplementary material for: Efficacy of aerobic exercise and a prudent diet for improving selected lipids and lipoproteins in adults: a meta-analysis of randomized controlled trials
Source: BMC Med. 2011 Jun 15;9:74. doi: 10.1186/1741-7015-9-74 (PMC3141539; doi:10.1186/1741-7015-9-74)

Study name	Subgroup within study	Point	Lower limit	Upper limit
Hellenius et al., 1993	none	-0.5	-4.7	3.7
McAuley et al., 2002	none	0.2	-3.2	3.6
Miller et al., 2002	none	0.1	-3.6	3.9
Nieman et al., 2002	none	-0.2	-4.4	4.0
Stefanick et al., 1998	men	-0.6	-4.4	3.2
Stefanick et al., 1998	women	-0.2	-4.0	3.5
Wood et al., 1991	men	-1.6	-4.0	0.8
Wood et al., 1991	women	-0.9	-5.0	3.2
		-0.5	-4.0	3.1

Difference in means (95% CI) with study removed

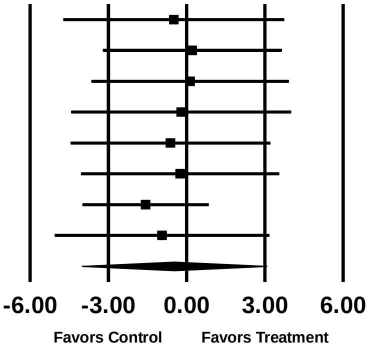

Supplement: Supplementary file 7 [file 1741-7015-9-74-S4.DOC]
