# Supplementary material for: Efficacy of aerobic exercise and a prudent diet for improving selected lipids and lipoproteins in adults: a meta-analysis of randomized controlled trials
Source: BMC Med. 2011 Jun 15;9:74. doi: 10.1186/1741-7015-9-74 (PMC3141539; doi:10.1186/1741-7015-9-74)

Study name	Subgroup within study	Point	Lower limit	Upper limit
Miller et al., 2002	none	-0.46	-0.71	-0.22
Nieman et al., 2002	none	-0.45	-0.71	-0.18
Stefanick et al., 1998	men	-0.43	-0.69	-0.17
Stefanick et al., 1998	women	-0.49	-0.74	-0.24
Wood et al., 1991	men	-0.33	-0.47	-0.19
Wood et al., 1991	women	-0.47	-0.76	-0.18
		-0.43	-0.65	-0.22

Difference in means (95% CI) with study removed

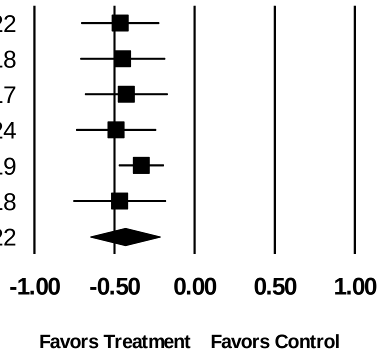

Supplement: Supplementary file 8 [file 1741-7015-9-74-S5.DOC]
