# Supplementary material for: Efficacy of aerobic exercise and a prudent diet for improving selected lipids and lipoproteins in adults: a meta-analysis of randomized controlled trials
Source: BMC Med. 2011 Jun 15;9:74. doi: 10.1186/1741-7015-9-74 (PMC3141539; doi:10.1186/1741-7015-9-74)

Study name	Subgroup within study	Point	Lower limit	Upper limit
Hellenius et al., 1993	none	-9.6	-13.7	-5.6
McAuley et al., 2002	none	-10.2	-13.7	-6.6
Miller et al., 2002	none	-8.7	-12.3	-5.2
Nieman et al., 2002	none	-8.2	-11.4	-4.9
Stefanick et al., 1998	men	-8.6	-12.3	-5.0
Stefanick et al., 1998	women	-9.0	-12.7	-5.3
Wood et al., 1991	men	-10.3	-13.8	-6.8
Wood et al., 1991	women	-9.4	-13.8	-4.9
		-9.2	-12.7	-5.8

Difference in means (95% CI) with study removed

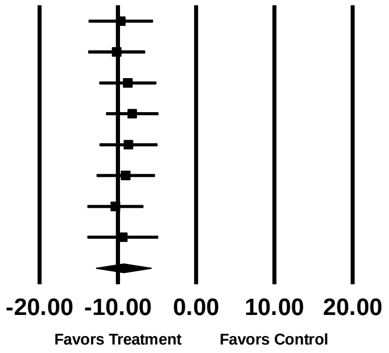

Supplement: Supplementary file 9 [file 1741-7015-9-74-S6.DOC]
