# Supplementary material for: Efficacy of aerobic exercise and a prudent diet for improving selected lipids and lipoproteins in adults: a meta-analysis of randomized controlled trials
Source: BMC Med. 2011 Jun 15;9:74. doi: 10.1186/1741-7015-9-74 (PMC3141539; doi:10.1186/1741-7015-9-74)

Study name	Subgroup within study	Point	Lower limit	Upper limit
Hellenius et al., 1993	none	-11.2	-19.1	-3.2
McAuley et al., 2002	none	-12.1	-19.7	-4.6
Miller et al., 2002	none	-12.1	-18.9	-5.2
Nieman et al., 2002	none	-9.0	-15.6	-2.5
Stefanick et al., 1998	men	-10.4	-17.1	-3.7
Stefanick et al., 1998	women	-10.5	-17.3	-3.6
Wood et al., 1991	men	-7.8	-13.7	-2.0
Wood et al., 1991	women	-11.3	-19.9	-2.6
		-10.6	-17.2	-4.0

Difference in means (95% CI) with study removed

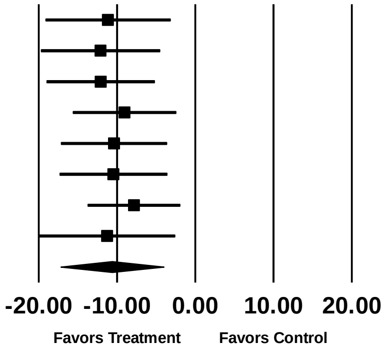

Supplement: Supplementary file 10 [file 1741-7015-9-74-S7.DOC]
