# Supplementary material for: Efficacy of aerobic exercise and a prudent diet for improving selected lipids and lipoproteins in adults: a meta-analysis of randomized controlled trials
Source: BMC Med. 2011 Jun 15;9:74. doi: 10.1186/1741-7015-9-74 (PMC3141539; doi:10.1186/1741-7015-9-74)

Study name	Subgroup within study	Cumulative statistics		
		**Point**	**Lower limit**	**Upper limit**
Wood et al., 1991	men	-9.3	-11.4	-7.2
Wood et al., 1991	women	-9.6	-10.7	-8.4
Hellenius et al., 1993	none	-10.3	-12.0	-8.6
Stefanick et al., 1998	men	-10.6	-12.5	-8.8
Stefanick et al., 1998	women	-10.9	-12.8	-9.0
McAuley et al., 2002	none	-12.4	-15.2	-9.7
Miller et al., 2002	none	-13.0	-15.8	-10.2
Nieman et al., 2002	none	-15.5	-20.3	-10.7
		-15.5	-20.3	-10.7

Cumulative difference in means (95% CI)

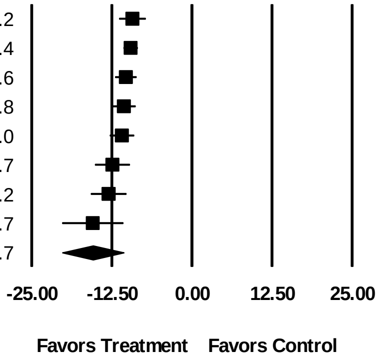

Supplement: Supplementary file 11 [file 1741-7015-9-74-S8.DOC]
