# Supplementary material for: Efficacy of aerobic exercise and a prudent diet for improving selected lipids and lipoproteins in adults: a meta-analysis of randomized controlled trials
Source: BMC Med. 2011 Jun 15;9:74. doi: 10.1186/1741-7015-9-74 (PMC3141539; doi:10.1186/1741-7015-9-74)

Study name	Subgroup within study	Cumulative statistics		
		**Point**	**Lower limit**	**Upper limit**
Wood et al., 1991	men	7.3	6.9	7.8
Wood et al., 1991	women	5.0	0.5	9.6
Hellenius et al., 1993	none	3.2	-1.4	7.9
Stefanick et al., 1998	men	2.6	-1.4	6.6
Stefanick et al., 1998	women	1.7	-1.9	5.3
McAuley et al., 2002	none	0.5	-4.0	5.1
Miller et al., 2002	none	-0.2	-4.4	4.0
Nieman et al., 2002	none	-0.5	-4.0	3.1
		-0.5	-4.0	3.1

Cumulative difference in means (95% CI)

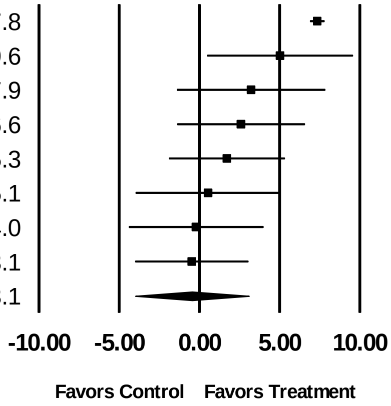

Supplement: Supplementary file 12 [file 1741-7015-9-74-S9.DOC]
